# Optimization of brain PET markerless head motion correction in a large human cohort

**DOI:** 10.1186/s40658-026-00885-1

**Published:** 2026-05-29

**Authors:** Tianyi Zeng, Jiazhen Zhang, Jean-Dominique Gallezot, Kathryn Fontaine, Weize Jiang, Paul Gravel, Tim Mulnix, Mika Naganawa, Zhaohui Yang, Xinyue Zhang, Su Wang, Xishan Sun, Lingzhi Hu, Richard E. Carson

**Affiliations:** 1 Department of Radiology and Biomedical Imaging, Yale University, New Haven, CT USA; 2 Department of Biomedical Engineering, Yale University, New Haven, CT USA; 3 United Imaging Healthcare, Houston, TX USA

**Keywords:** Markerless motion tracking system, Brain PET, Head motion, Event-by-event correction

## Abstract

**Purpose:**

Uncorrected head motion in brain PET degrades image resolution and biases quantification. Previously, we evaluated the United Imaging Healthcare markerless motion tracking (UMT) system for brain PET (Baseline UMT). This study provides an expanded validation in a large cohort compared to the Vicra with improved methodology (Optimized UMT).

**Methods:**

We developed automatic processing to define the upper face region and added a 2-step Iterative Closest Points registration to reduce non-rigid motion effects. Optimized and Baseline UMT were first compared with simulations using measured motion and intentional facial expressions. In the human study, we evaluated 51 dynamic human studies (Siemens mCT) using 5 radiopharmaceuticals. Vicra data were used as the gold standard, after assessment of motion data quality using motion-corrected centroid-of-distribution. Comparisons between Optimized UMT, no motion correction (NMC), Post-Reconstruction Registration (PRR), and Baseline UMT were performed. Static and dynamic analyses assessed the differences of region-of-interest standardized uptake values (SUV) compared to Vicra. Time activity curve (TAC) analysis was performed to quantify uncorrected motion from the residual standard deviation (SD) around fitted TACs.

**Results:**

In simulation studies, optimized UMT reduced error by 20% relative to NMC and by 9% relative to Baseline UMT under intentional facial expressions, reflecting the impact of the updated UMT registration. In the human static analysis of SUV, optimized UMT outperformed PRR (+ 1.6%) and NMC (+ 6.5%) on average grey matter difference. Similar results were found in the dynamic analysis. In the TAC analysis, optimized UMT consistently yielded lower residual variability than NMC and PRR across tracers and ROIs, with a narrower range of median %SD values (0.3–1.8%) compared with NMC (1.6–6.5%) and PRR (0.5–1.9%), while demonstrating performance comparable to the reference Vicra-based correction.

**Conclusion:**

Optimized UMT achieved comparable performance to Vicra and significantly improved quantification over PRR.

**Supplementary Information:**

The online version contains supplementary material available at 10.1186/s40658-026-00885-1.

## Introduction

Positron emission tomography (PET) has been extensively employed for human brain imaging due to the availability of a wide range of specific radiotracers. Recently, the ultra-high performance brain PET system – NeuroEXPLORER (NX) was developed, offering high sensitivity and excellent spatial resolution [1, 2]. However, exceptional brain image quality cannot be achieved without highly accurate motion correction (MC). Uncorrected head motion during PET imaging reduces image resolution, impacts tracer distribution estimation, and introduces attenuation correction (AC) mismatch artifacts [3]. Current techniques for MC include data-driven and hardware-based motion tracking (HMT). Data-driven methods utilize image registration or PET raw data. Image registration-based MC typically uses pre-defined dynamic frames registered to a reference frame to estimate motion [4]. However, these methods do not address intraframe motion and can suffer from attenuation mismatch. To reduce intraframe motion effects, Spangler-Bickell et al. [5] developed ultrafast reconstruction techniques to estimate motion from short-duration frames in brain PET imaging. This approach used intensity-based image registration methods for frame alignment, which is sensitive to varying count level and often necessitates manual parameter adjustments. Other data-driven methods include centroid-of-distribution (COD) approaches and deep learning methods, which estimate and subsequently correct head motion from raw PET data. COD-based methods register images reconstructed from periods of minimal motion (“motion-free frames”) and the accuracy of the registration is dependent on the duration and count level of these frames [6]. Current deep learning-based data-driven methods have shown good performance, but they necessitate tuning for each tracer and may be ineffective in cases of significant changes in tracer distribution, e.g., at early times postinjection [7–10].

An alternative approach involves using external hardware devices for motion tracking, either using marker-based or markerless HMT. Because motion estimates are derived from external measurements rather than PET count statistics or reconstructed images, these approaches can robustly capture and correct rapid head motion and are inherently independent of activity distribution, tracer kinetics and counting statistics [3]. Marker-based HMT, such as Polaris Vicra (Northern Digital Inc., referred to as “Vicra”) [11, 12], Polaris Vega [13] and electromagnetic motion tracking (EMMT) [14], requires attaching markers on the patient’s head, introducing potential tracking errors due to relative motion between the head and the marker. At the Yale PET Center, over 5,000 Vicra motion-corrected PET studies have been conducted, revealing that tool slippage and wobbling can adversely affect the accuracy of Vicra motion detection [15]. Therefore, an ideal HMT method should be robust and markerless.

Feasibility of markerless motion tracking during PET scanning has been evaluated and several different markerless motion tracking systems have been applied in human brain imaging [16–18]. Previously, we performed an initial evaluation of a markerless head motion tracking device, the United Imaging Healthcare markerless motion tracking system (UMT). That study compared UMT MC to that of the Polaris Vicra, used as the gold standard, for Siemens mCT PET/CT studies using event-by-event (EBE) MC [15]. The UMT system utilizes a stereovision camera that employs infrared structured light to capture the 3D facial surface of a patient at a frame rate of 30 Hz. To determine motion vectors, the point cloud of each frame is registered with a reference frame using an iterative closest point (ICP) registration algorithm. The motion tracking information is then incorporated into the Motion-compensation OSEM List-mode Algorithm for Resolution-recovery reconstruction (MOLAR) [19]. In this previous study, the segmentation task (definition of the portion of the face for registration) was manually performed for the reference frame. This is suboptimal due to the diversity of facial features, making appropriate segmentation challenging, operator-dependent, and less reproducible. Moreover, non-rigid motion and/or large-scale rigid motion can cause discrepancies between the moving and reference frames, impacting the accuracy of ICP and the final motion tracking accuracy [15]. Furthermore, the prior study only compared UMT-based EBE MC to the Vicra marker-based HMT method and did not also compare it with the commonly used post-reconstruction registration (PRR) MC method [20].

In this paper, we optimized the UMT motion tracking methodology by implementing automatic face segmentation and improved the UMT point cloud registration method to address non-rigid face motion. Importantly, we pre-assessed the quality of the Vicra motion data, in order to only use high-quality data as the gold standard. We assessed the effectiveness of the optimized UMT method through comprehensive studies, including simulation study with intentional motion and a large human cohort study comprising 51 human scans with 5 different tracers on the Siemens mCT. We compared the optimized UMT MC with the previous UMT MC (“Baseline UMT”), PRR and Vicra methods. This comparison involved comparison of brain region-of-interest (ROI) values, as well as overall assessment of MC quality by examining residual variation around time-activity curves (TACs). Furthermore, we tested the proposed UMT MC method in the NeuroEXPLORER ultra-high-performance brain PET system.

## Materials and methods

### Optimization of markerless motion tracking

To enhance the efficacy of the UMT, we optimized the motion tracking method described in [15]. In the previous approach, a bounding box was manually selected and applied to the reference cloud to include the upper face/cheek area and exclude all non-face areas. Here, additional automatic processing was applied to the UMT reference point cloud to define the bounding box. Due to the oblique angle of the UMT camera with respect to the face, there will be “holes” in the face, e.g., the eye sockets. A morphological closing operator was utilized to fill the incomplete regions in the point cloud and complete the facial area (see Supplemental material and Supplemental Fig. 1). Based on the location of the camera, we determined a rough estimate of the subject’s head location. Within this region, a bounding box surrounding the face was defined based on assuming that the face represents the largest connected component. Initial experiments showed that the chin region affected registration accuracy, so we automatically reduced the axial extent of the bounding box by 20% (determined empirically).

**Fig. 1:**
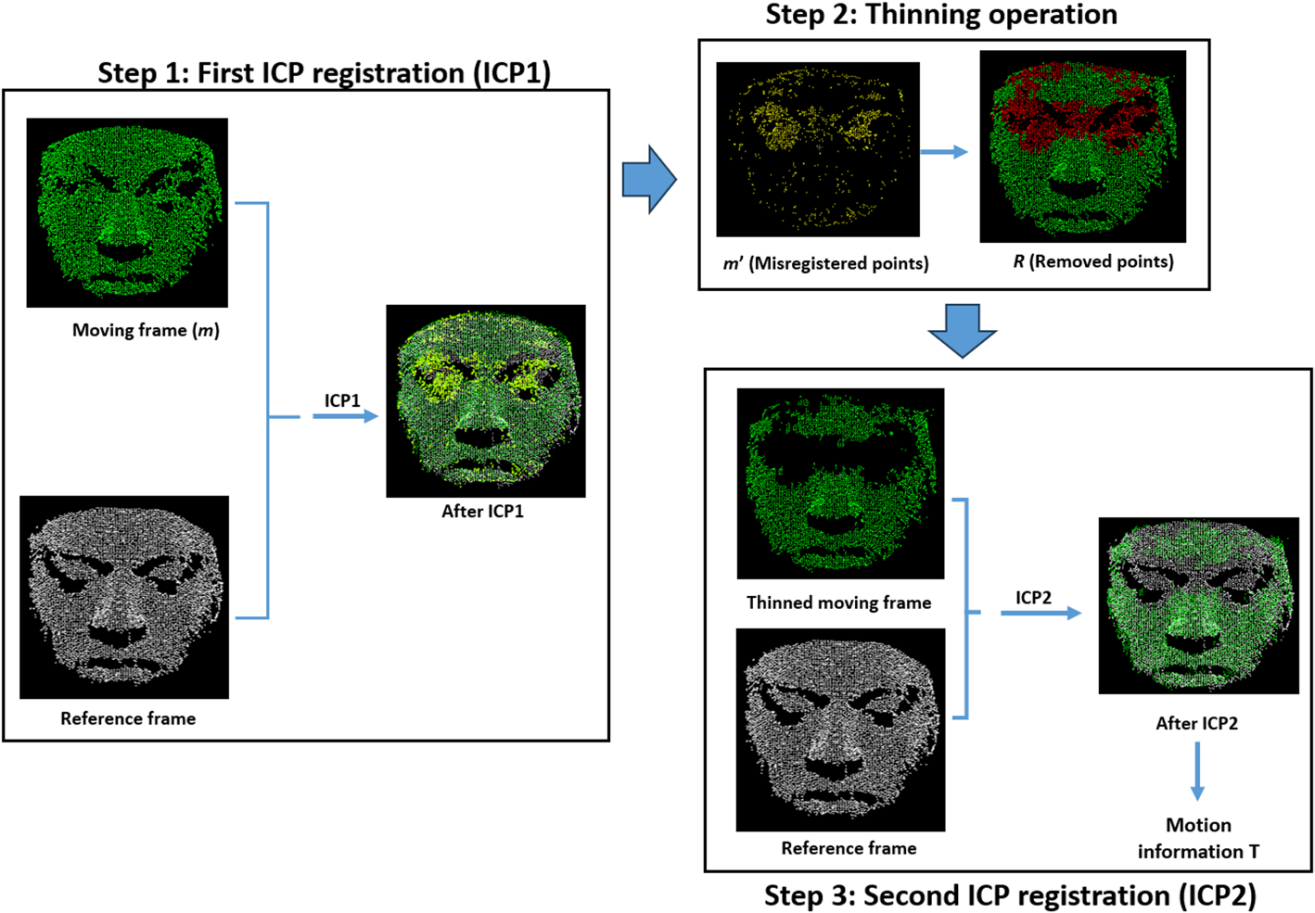
Flow chart of the updated UMT registration method using an example with eyebrow movement. White points represent the reference point cloud and green points represent the moving point cloud. Step 1 describes the first ICP registration, and Step 2 describes the thinning of the moving frame to address facial expression changes. The yellow points in Step 1 (after ICP1) and Step 2 indicate the portion of the moving frame with misregistration to the reference frame after the first ICP registration. The red points in Step 2 indicate the portion of moving frame removed for secondary ICP (Step 3). See text for details. ICP: iterative closest point

In addition, the process of registration of point clouds from a moving frame to the reference frame was updated (Fig. 1). In Step 1, the point-to-plane ICP registration algorithm [21] was implemented within the bounding box and the set of misregistered points ${m}^{{\prime }}$ was determined as follows:

1 $$ m^{\prime } \in \left\{ {m_{i} |\frac{{\overrightarrow {{v_{i} }} \cdot \overrightarrow {{n_{i} }} }}{{\left\| {\overrightarrow {{n_{i} }} } \right\|}} \ge d} \right\} $$

where * m* represents the set of registered moving frame points within the reference frame bounding box, and * i* indexes the points in the point cloud. The vector between pairs of moving and reference points is denoted as $\overrightarrow{{v}_{\mathrm{i}}}$, and $\overrightarrow{{n}_{\mathrm{i}}}$ is the normal vector of the reference surface plane at point *i*. $\frac{{\overrightarrow {{v_{{\mathrm{i}}} }} \cdot \overrightarrow {{n_{{\mathrm{i}}} }} }}{{\left\| {\overrightarrow {{n_{{\mathrm{i}}} }} } \right\|}}$ is the distance of moving point *i* (after registration) to the reference surface plane; this formulation matches the distance calculation method of the point-to-plane ICP algorithm. The threshold * d* to define misregistered points was empirically set to 1.0 mm. To address facial expression changes, e.g., eyebrow movements, which may lead to motion misestimation, we performed a thinning operation to eliminate these points from the moving frame. As shown in Step 2 of Fig. 1, $m^{\prime }$ was down-sampled by averaging point locations within a 3 mm voxel grid [22], and the largest contiguous set of misregistered points in $m^{\prime }$ was determined using Breadth First Search (BFS) algorithm [23] and all points within this set were removed (*R* in Fig. 1, Step 2). Then, a second ICP registration was performed (Fig. 1, Step 3), excluding the points in * R*. These automated improvements led to improved registration accuracy (see Results).

### Human data

All experiments were conducted on a Siemens Biograph mCT scanner [24], with both Vicra and UMT employed for motion tracking. The UMT was mounted on the gantry of the scanner, and spatial calibration and time synchronization were applied between UMT and mCT following the same procedure in [15]. The goal was to assess UMT results, using Vicra as the gold standard.

A total of 59 PET dynamic human studies were undertaken. Before using the Vicra data as a gold standard, we assessed the accuracy of the Vicra motion information using motion corrected centroid-of-distribution (MCCOD) by eliminating motion data of insufficient quality [25] (see Supplemental material and Supplemental Fig. 2). Eight of these studies were excluded because of displacements in the Vicra MCCOD trace representing uncorrected motion, so 51 studies were analyzed.

Eighteen 90-min PET dynamic studies were performed using [^11^C]LSN3172176 [26] (572 ± 171 MBq, muscarinic M1 receptor, *N* = 18). Twenty-three 120-min PET dynamic studies were performed using radiotracers including [^18^F]Bavarostat [27] (135 ± 49 MBq, HDAC6 enzyme, *N* = 14), and [^18^F]FPEB [28] (138 ± 46 MBq, metabotropic glutamate 5 receptors, *N* = 9). Seven 30-min PET dynamic studies (90–120 min post-injection) were performed using [^18^F]NCFHEB [29] (156 ± 48 MBq, nicotinic acetylcholine α4β2 receptor, *N* = 7). Three 90-min PET dynamic studies were performed using [^11^C]PBR28 [30] (441 ± 98 MBq, neuroinflammation marker/translocator protein (TSPO), *N* = 3). Computed tomography (CT) scans acquired before the PET were utilized for AC, and each participant underwent a separate T1-weighted MRI scan.

### Simulated data

To investigate the impact of large rigid motions and facial expressions on ROI quantification, two simulations were performed using Vicra and UMT motion data. The ground truth activity image for the simulation was derived from one human subject, specifically the MR image and the corresponding attenuation image. The MR image was acquired using a 3D T1-weighted MPRAGE sequence on a 3T Siemens Prisma Fit scanner (Siemens Healthcare, Erlangen, Germany) with the following parameters: TR = 2530 ms, TE = 2.81 ms, TI = 1100 ms, and flip angle = 7°. The grey matter (GM) and white matter (WM) regions were segmented by Computational Anatomy Toolbox (CAT) from the MR image and the activity image was created with a GM: WM contrast of 4:1 (Supplemental Fig. 3). Listmode data were simulated using the forward projection model of MOLAR [11] for a 5-min scan based on the mCT scanner geometry [19]. Four different listmode files were created with different Vicra motion files used as the ground truth. The selection of motion files was guided by motion magnitude calculations (see Supplement for details). For the first simulation focusing on rigid motions, a video based on the UMT data in the selected frames was reviewed (see Supplemental material and Supplemental Fig. 4). No conspicuous facial expressions were discernible in the UMT video during these periods.

A second simulation was performed to investigate the effects of facial expressions on UMT motion tracking. To collect the needed motion data, two volunteers each participated in two 5-minute intentional facial expression experiments conducted with Vicra and UMT data collection with no tracer injection. The volunteers were instructed to perform intentional facial expressions including mouth breathing, talking, eyebrow movements, and eye rubbing, while also performing continuous rigid motion (see Supplement for details). Subsequently, four 5-min listmode files including these motions were simulated, using the Vicra data as the ground truth. See Supplement for details of the simulation methodology.

### Event-by-event motion compensated reconstruction

Image reconstruction for simulated and human data was performed using the MOLAR platform [19] following the same workflow in [15]. Rigid motion information at 30 Hz provided by Vicra or UMT was produced in the same format. OSEM reconstruction (3 iterations × 21 subsets) with spatially invariant point-spread-function of 4 mm full-width-half-maximum (FWHM) was used, with 2.0-mm isotropic voxels and 200 × 200 × 111 image size for simulation and human studies. Time-of-flight information was included in the reconstruction and no post-smoothing was applied. For simulation data, static reconstruction was performed without scatter and random corrections. For human data, all standard physics corrections were applied, including attenuation, normalization, scatter, randoms, and decay. Dynamic 120-min PET data ([^18^F]Bavarostat, [^18^F]FPEB, and [^11^C]PBR28) were reconstructed into 33 frames: 6 × 30 s, 3 × 1 min, 2 × 2 min, 22 × 5 min. For 90-min human data ([^11^C]LSN3172176), the reconstruction used 16 × 5 min frames. For 30-min scans ([^18^F]NCFHEB), data were reconstructed into 6 × 5 min frames.

Several MC methods were applied in the simulation and human including: (i) no motion correction (NMC), (ii) Post Reconstruction Registration (PRR), which utilized a mutual information-based similarity metric to rigidly register NMC images using the FLIRT toolkit [31]; (iii) the Baseline UMT method [15]. (iv) the Optimized UMT method presented above, and (v) Vicra method, considered as the gold standard.

### Image analysis

For the simulation study, the ground truth activity image (Supplemental Fig. 3) was registered to reconstructions with different motion corrections utilizing BioImage Suite (rigid registration with normalized mutual information metric). In each case, the mean GM and WM values were determined, using GM and WM masks that were eroded (structuring element set to 2 mm) to reduce the partial volume effect. The activity ratio of GM to WM was calculated and expressed with respect to the reference Vicra GM: WM ratio as a percent GM: WM ratio (PGWR). A higher PGWR indicated better MC.

For each human study, the PET image from 0 to 10 min (90–100 min for [^18^F]NCFHEB) was coregistered to each subject’s T1-weighted MRI image via a rigid registration with mutual information [31]. The individual MR image was then coregistered to the Montreal Neurological Institute space using an affine linear plus nonlinear registration (BioImage Suite), to apply ROIs from the automated anatomic labeling (AAL) template to the PET images [32]. The 66 GM regions selected from the AAL template were refined by applying a binary GM mask generated by thresholding the CAT-derived gray-matter tissue probability map at 0.5 (GM probability > 50%) in the subject MR space [33].

For static analysis of the human scans (average of entire scan), the ROIs were merged into eleven large GM ROIs: amygdala, caudate nucleus, cerebellum cortex, frontal cortex, hippocampus, insula, occipital cortex, parietal cortex, putamen, temporal cortex, and thalamus. Standardized uptake values (SUVs) were extracted for each MC approach, and the percent differences compared to the reference Vicra-corrected reconstruction were calculated. For dynamic analysis, the 66 GM ROI values were extracted, the percent differences compared to Vicra were calculated, and the mean and standard deviation (SD) of the percent differences were tabulated across all subjects for all ROIs. Data from the first minute of the scan was excluded due to highly variable results for PRR.

In addition to assessing ROI bias, residual motion effects on variability in TACs of specific ROIs (selected for each tracer) were calculated. For each subject, ROI, and MC method, a 3rd order polynomial fit was applied to TAC data from 30-90-minute post-injection. The residuals between the measured TAC and the fitted curve were then computed, and their variability was quantified using the residual SD. This residual SD was normalized by the mean value of the fitted TAC over the same time window and expressed as a percentage. We then assessed whether one method had a significantly smaller variance than the other methods using a single-tail Wilcoxon signed-rank test (*p* < 0.05). [^18^F]NCFHEB scans were excluded from this analysis due to their shorter scan duration of 30 min.

## Results

### Simulation study

Table 1 presents the results of PGWR analysis derived from the simulation study. In the large rigid motion study (simulation 1), NMC exhibited lower PGWR (88.4%) due to the presence of rigid motion. Both the Baseline UMT and Optimized UMT methods yielded higher PGWR values, with the Optimized UMT demonstrating slightly higher values with smaller SD. In the intentional facial expression study (simulation 2), the PGWR of NMC dropped to 63.9%, while the Baseline UMT produced a moderate improvement (75.0%), indicating remaining errors due to the ICP rigid registration. The Optimized UMT showed an improvement over the Baseline UMT in mean PGWR (8.9% higher) and a smaller SD (6.8% lower). However, further improvement is needed to handle substantial facial expressions (see Discussion).

### Human PET static study

Table 2 shows the SUV analysis for [^11^C]LSN3172176 averaging over the entire scan duration, focusing on large GM ROIs using NMC, PRR, Baseline UMT, and Optimized UMT. The mean and SD of the percent differences were tabulated across all subjects for all ROIs and the percentage differences relative to the Vicra were reported. SUVs were first averaged across the eleven GM ROIs for each subject to obtain a subject-level GM SUV, after which percent differences relative to Vicra were computed and summarized across subjects to derive the GM Average and GM SD. Optimized UMT yielded a slightly higher GM Average compared to Vicra, with a small SD (0.6% ± 0.9%). Conversely, NMC exhibited a notable negative GM Average bias and a large GM SD (-6.9% ± 6.5%). When compared to other MC methods, Optimized UMT demonstrated an average enhancement of 1.9% over PRR and 2.2% over Baseline UMT. The Optimized UMT also exhibited the lowest GM SD among all methods. For the other tracers, static SUV analysis results over the entire scan duration can be found in the Supplemental Table 1. Across all 51 scans, Optimized UMT consistently outperformed PRR (+ 1.6% on average) and NMC (+ 6.5% on average) in terms of GM Average difference. For these other tracers, Optimized and Baseline UMT had very similar results.

### Human PET dynamic studies

**Fig. 2:**
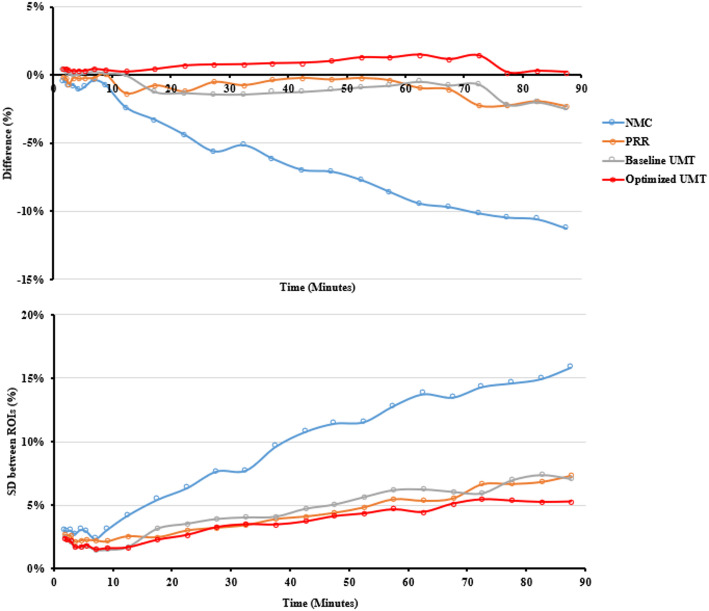
Mean (top) and SD (bottom) across small ROIs of the percent difference of NMC and 3 MC methods versus Vicra over individual scan frames for the human [^11^C]LSN3172176 study (*n* = 18). NMC: No Motion Correction. PRR: Post-reconstruction registration. SD: Standard deviation. UMT: United Imaging Healthcare Motion Tracking system

Figure 2 illustrates the dynamic SUV analysis conducted for [^11^C]LSN3172176 across 66 GM ROIs. In Fig. 2 (top), the mean difference between Optimized UMT and Vicra was + 0.7%, while NMC exhibited larger negative biases (-5.2%). Notably, the PRR curve (orange) had a mean bias of -0.8%. Furthermore, variability between subjects averaged over time for Optimized UMT was smaller (1.1%) than Baseline UMT (5.3%), PRR (3.0%) and NMC (5.7%). In Fig. 2 (bottom), the across-ROI SD of the percentage differences was low for all three MC methods throughout the entire scan period. Optimized UMT yielded the smallest average SD (3.3%), compared with Baseline UMT (4.0%), PRR (3.9%) and NMC (8.2%). Similar observations were noted for the other tracers (Supplemental Fig. 5).

**Fig. 3:**
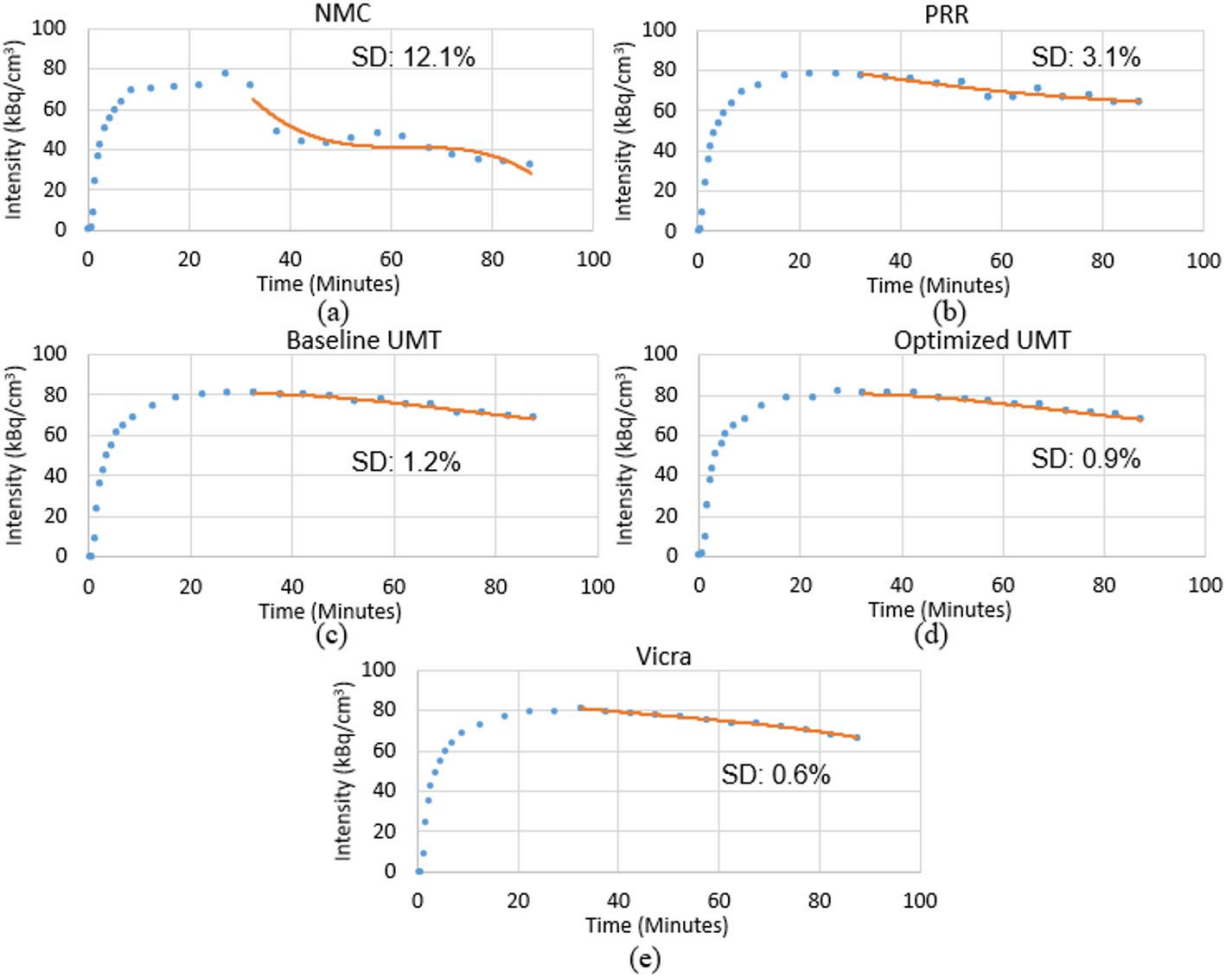
Example of caudate (volume 7.5 mL) TAC and fitting of data with different motion corrections for a [^11^C]LSN3172176 scan with substantial motion. The percent SD of the residuals around the fitting curve indicates higher variability in the results of NMC (**a**), PRR (**b**) and Baseline UMT (**c**) compared to Optimized UMT (**d**) and Vicra (**e**). NMC: No Motion Correction. PRR: Post-reconstruction registration. SD: standard deviation. TAC: time activity curve. UMT: United Imaging Healthcare Motion Tracking system

Figure 3 illustrates the TAC plot of the caudate with different MC methods from a [^11^C]LSN3172176 scan with substantial motion. Figure 3a shows the large deviation in the caudate TAC for the NMC data. In Fig. 3b, the PRR plot shows substantial improvement, but, in comparison to the UMT methods (Fig. 3c and d), has higher variability around the mean TAC curve. In this case, Optimized UMT achieved a lower %SD (0.9%) compared to Baseline UMT (1.2%) and was closer to the Vicra value (0.6%). Supplemental Fig. 6 presents TAC analysis from two additional [^11^C]LSN3172176 scans, a typical case with small motion which was corrected well by all methods and an extreme motion case. For the latter scan, PRR and Baseline UMT performed poorly due to large rigid motions and facial expressions. Both methods yielded biased ROI values and a significantly higher %SD than Vicra and Optimized UMT. Table 3 summarizes the residual %SD around the TAC fits for specific ROIs for each tracer, using different motion corrections. The results are shown as the median and interquartile range (difference between 75th and 25th percentiles), as opposed to mean and SD, because the values had a non-normal distribution. Across all comparisons, Optimized UMT consistently yielded smaller residual SD compared to NMC and PRR, exhibiting a narrower range of median %SD values (0.3–1.8%) compared with NMC (1.6–6.5%) and PRR (0.5–1.9%). Statistical analysis using Wilcoxon signed-rank test revealed that both NMC and PRR exhibited significantly higher TAC variability compared to Vicra (*p* < 0.05). Optimized UMT had very similar performance to Vicra, with no statistically significant increase in variability compared to Vicra except for the caudate for [^18^F]Bavarostat. Additionally, PRR had a significantly higher residual %SD compared to Optimized UMT for 4/6 ROIs tested.

## Discussion

In this study, we used a camera-based system (UMT) for head MC in dynamic PET. First, we optimized the UMT method with several technical innovations, including incorporating automatic face segmentation and improved point cloud registration, to mitigate the effects of non-rigid motion. We then compared the Optimized UMT with NMC, PRR, and Baseline UMT, using Vicra data as the gold standard through both simulations and a large human cohort study involving 51 subjects and 5 different radiopharmaceuticals. To the best of our knowledge, this represents the largest cohort for human brain markerless motion tracking evaluation to date. This large-scale evaluation enabled systematic identification and quantification of cases strongly affected by motion—something smaller studies could not adequately capture—and ensured the robustness of performance assessment under realistic clinical conditions.

The results demonstrated that the Optimized UMT outperformed all other methods, yielding results closely aligned with the gold standard. Compared with PRR, a MC method widely used routinely, the Optimized UMT enhanced the GM Average SUV values by 1.9% for [^11^C]LSN3172176, 1.9% for [^18^F]Bavarostat, 1.7% for [^18^F]FPEB, 0.3% for [^18^F]NCFHEB, and 0.3% for [^11^C]PBR28 in the human static study. Furthermore, we introduced a novel evaluation metric-variability of TAC fitting residuals to quantitatively assess the performance of different MC methods. This metric provides a direct measure of residual motion effects in dynamic PET. In this analysis, PRR exhibited higher TAC variability than the Optimized UMT, indicating a performance degradation in MC. Overall, we demonstrated that Optimized UMT significantly improved MC accuracy compared with PRR, underscoring the value of EBE MC with accurate HMT data and its potential to enhance current clinical and research PET workflows.

PRR suffered from attenuation mismatch and intra-frame motion effect, which have been the focus of recent improvements in state-of-the-art data-driven methods [34, 35]. In future work, we will compare the proposed UMT method with more advanced data-driven MC approaches, including systematic evaluation of their performance as a function of motion magnitude. However, unlike hardware-based tracking [36], data-driven approaches are fundamentally limited by PET counting statistics and tracer-dependent contrast, making them particularly vulnerable during early dynamic frames and in late low-count frames of [^11^C] tracers. These issues become more significant for data-driven methods that divide frames into sub-frames [5], with potential for introducing low-count bias.

It is important to note that we pre-assessed the quality of the Vicra data using MCCOD and removed 8 of 59 potential datasets due to sub-optimal Vicra MC, perhaps caused by marker tool slippage and wobbling. This was an improvement compared to our first study [15]. In that study, the Baseline UMT GM values for [^11^C]LSN3172176 exceeded Vicra values by 1.2%. Here, with higher-quality Vicra data, Baseline UMT underestimated GM Average by 1.6%, but the Optimized UMT yielded a higher GM Average by 0.6%. Over all 51 studies (11 ROIs each), the % difference between Optimized UMT and Vicra in the static ROI analysis was 0.04% ± 2.41%. These results emphasize the performance enhancement of the optimized UMT compared to Baseline UMT, which may provide help for quantitative imaging of clinical human scans.

In the simulation study (Table 1), we compared Optimized UMT with Baseline UMT under two different motion scenarios. Both methods corrected large rigid motions well, but facial expressions produced bias, with Baseline UMT more affected than Optimized UMT. These findings demonstrated that our optimization enabled UMT to provide more accurate data, although there is clearly room for further improvement for complex facial expressions. Future work will focus on developing data rejection and filtering strategies to mitigate the influence of strong non-rigid facial motion to enhance overall tracking robustness. A related example was found in the human study (Supplemental Fig. 6b), where Baseline UMT failed to accurately track extreme motions, causing TAC inaccuracy. In the human static analysis, optimized UMT achieved slightly higher GM Average SUV than the Vicra for 4 of 5 tracers, ranging from 0.1 to 0.6%. The exception was [^18^F]FPEB with a mean difference of -0.9%. This was caused by one subject where optimized UMT had a GM Average difference of -7.5% compared to Vicra. For this subject, the UMT video showed that the subject moved out of the UMT field-of-view multiple times and their hands obstructed the UMT during sneezing episodes. To address this class of motions, we plan to evaluate a real-time UMT intrinsic metric [15] to allow removal of data from periods of uncertain motion data.

While the variability of TAC fitting residuals provides a sensitive and practical metric for comparing motion-correction performance in dynamic scans, it is important to recognize that this measure reflects not only residual motion but also intrinsic statistical noise in the PET measurements. Frame-level noise depends on tracer kinetics, regional uptake, ROI size, and counting statistics, and therefore differs across tracers and brain regions. Although these noise contributions are identical across motion-correction methods for a given dataset, they nonetheless impose a fundamental lower bound on the achievable TAC variability. In future work, we will explicitly quantify this intrinsic noise floor by generating multiple statistically independent realizations of the list-mode data through controlled undersampling and computing the across-replicate %SD of the resulting TACs. This tracer and ROI-specific intrinsic noise will establish a quantitative benchmark against which the ultimate accuracy and limits of motion-correction methods can be assessed.

**Fig. 4:**
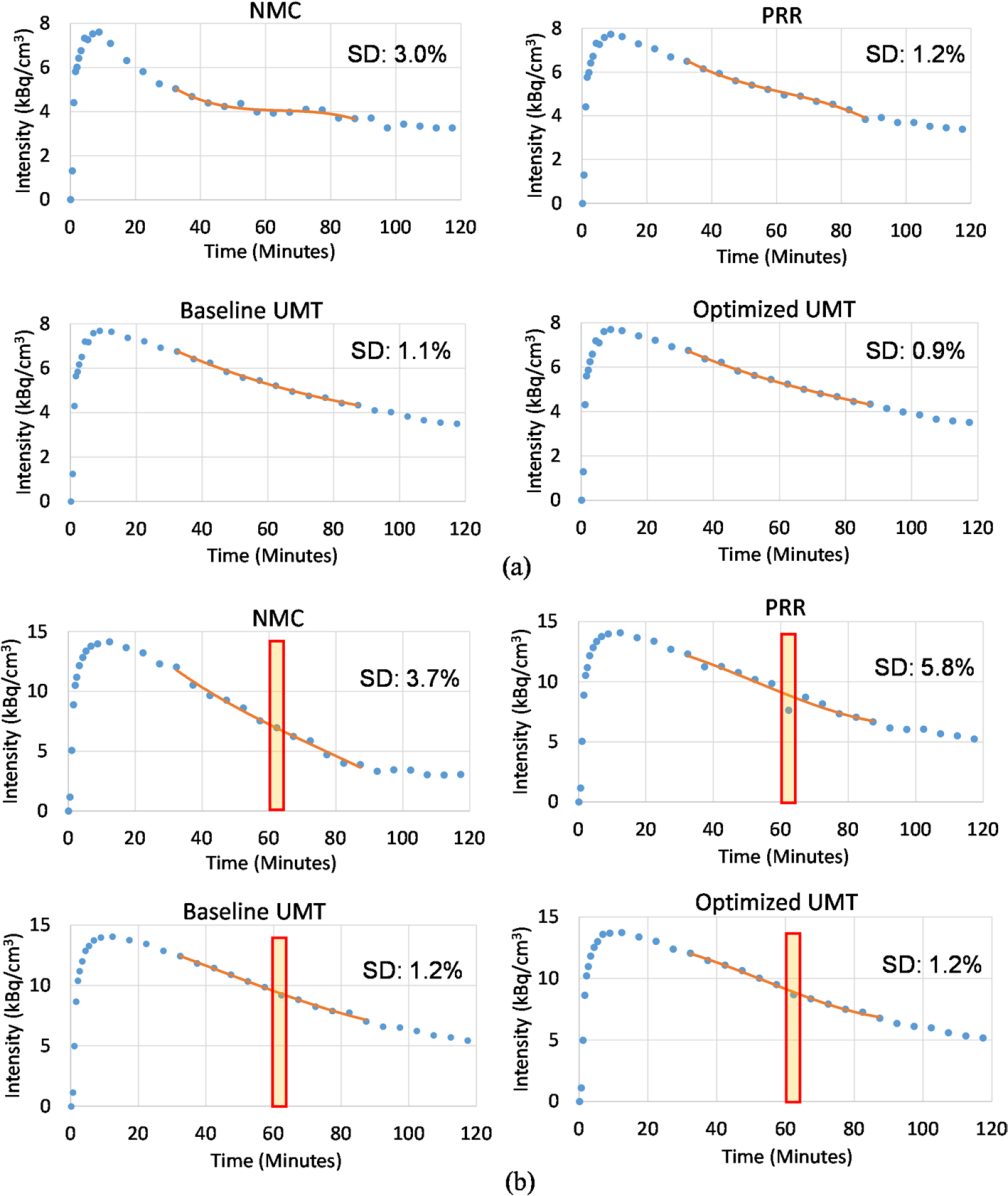
Examples of caudate (volume 6.4 mL) TAC and fitting of data with different motion corrections for two [^18^F]FE-PE2I NeuroEXPLORER scans with moderate motion (**a**) and large motion (**b**). In (**b**), a large motion occurred midway through the 60–65 min frame, causing noticeable bias in PRR. The percent SD of the residuals around the fitting curve indicates higher variability for NMC and PRR. NMC: No Motion Correction. PRR: Post-reconstruction registration. SD: standard deviation. TAC: time activity curve. UMT: United Imaging Healthcare Motion Tracking system

The ultimate goal of the UMT system is to apply it to ultra-high-performance systems such as the NX [1]. Here, we present data from two healthy control studies with the dopamine transporter ligand [^18^F]FE-PE2I [37]: one with moderate motion and one with large motion. Figure 4 shows the TAC analysis for the caudate for these cases and Table 4 summarizes the residual SD around the TAC fits for specific ROIs using different motion corrections. The Vicra was not used in the NX scans, and the UMT motion data are shown in Supplemental Fig. 7. For the moderate motion case (Fig. 4a), the MC methods performed in a similar manner to the mCT data, i.e., substantial improvements over NMC, with UMT showing slightly better performance than PRR. For the large motion case (Fig. 4b), the subject moved substantially at ~ 62 min, midway through one of the 5-min frames (highlighted in Fig. 4b), as shown from the UMT motion data (Supplemental Fig. 7b). In this case, the PRR TAC shows higher SD caused by an outlier frame (60–65 min postinjection) due to the intraframe motion. For both cases, Optimized UMT shows a smaller Striatum Average than NMC and PRR. Evaluation of MC accuracy in a large cohort of NX datasets is currently ongoing to assess the relative performance of UMT versus PRR in studies with focal uptake of targeted radiopharmaceuticals.

## Conclusion

We optimized the performance of the markerless UMT camera system and evaluated its performance with simulation and a large human cohort study on the mCT. Optimized UMT achieved comparable performance to gold standard Vicra, and significantly improved quantification accuracy compared to PRR and the Baseline UMT method. Ongoing work includes evaluation in human studies on the NX and further refinements to minimize effects of facial expressions on MC.

**Table 1:** Percent grey matter : white matter ratio (PGWR) (mean ± SD) analysis for the simulation study

MC methods	Simulation 1: Large rigid motion (*n* = 4)	Simulation 2: Intentional facial expressions (*n* = 4)
NMC	88.4% ± 1.0%	63.9% ± 4.8%
Baseline UMT	99.4% ± 1.1%	75.0% ± 12.9%
Optimized UMT	99.6% ± 0.4%	83.9% ± 6.1%

No Motion Correction (NMC) and UIH motion tracking (UMT) errors indicate the average ± standard deviation of percent GW:WM ratio as a percentage of the Vicra GM:WM ratio. These results are derived from 8 simulated listmode files: large rigid motions (*n* = 4) and intentional facial expressions (*n* = 4). GM: grey matter. WM: white matter

**Table 2:** Bias in static brain ROI analysis of [^11^C]LSN3172176 studies (*n* = 18)

Tracer	[^11^C]LSN3172176				
Brain ROI	NMC	PRR	Baseline UMT	Optimized UMT	
Amygdala	-3.1% ± 7.0%	-1.3% ± 3.8%	-1.4% ± 5.0%	-0.2% ± 1.3%	
Caudate	-15.4% ± 15.8%	-3.8% ± 5.7%	-3.4% ± 12.1%	-0.4% ± 1.3%	
Cerebellum cortex	-0.9% ± 11.5%	-1.2% ± 6.0%	0.8% ± 5.5%	-0.8% ± 4.3%	
Frontal	-11.9% ± 10.1%	-0.8% ± 3.3%	-0.8% ± 6.4%	0.7% ± 1.1%	
Hippocampus	-1.4% ± 7.3%	-0.3% ± 2.4%	-1.1% ± 4.8%	0.4% ± 2.4%	
Insula	-4.4% ± 4.2%	-0.8% ± 2.4%	-0.5% ± 4.1%	0.5% ± 0.7%	
Occipital	-2.3% ± 4.5%	0.3% ± 5.4%	-2.1% ± 14.1%	1.9% ± 3.7%	
Parietal	-5.2% ± 8.5%	-0.9% ± 5.6%	-2.5% ± 12.8%	1.5% ± 2.7%	
Putamen	-10.4% ± 9.7%	-2.1% ± 5.2%	-2.0% ± 10.0%	0.6% ± 0.9%	
Temporal	-7.1% ± 4.4%	-1.3% ± 3.2%	-1.2% ± 7.9%	0.7% ± 1.4%	
Thalamus	-2.4% ± 2.8%	-0.1% ± 1.0%	-0.5% ± 2.5%	0.3% ± 1.1%	
GM Average ± SD	-6.9% ± 6.5%	-1.3% ± 3.5%	-1.6% ± 8.0%	0.6% ± 0.9%	

ROI bias values shown as average ± standard deviation of the % difference with respect to Vicra values across all subjects. To calculate GM Average and GM SD, SUVs were first averaged across all GM ROIs for each subject to obtain a subject-level GM SUV. Percent differences relative to Vicra were then calculated at the subject level and summarized across subjects. NMC: No Motion Correction. PRR: Post-reconstruction registration. ROI: region-of-interest. SD: standard deviation. SUV: Standardized Uptake Values. UMT: United Imaging Healthcare Motion Tracking system

**Table 3:** Time-activity curve fitting residual percent SD

Tracer	Brain ROI	NMC	PRR	Baseline UMT	Optimized UMT	Vicra
[^11^C]LSN3172176 (*n* = 18)	Caudate	3.5%(3.8%)*†	1.6%(1.2%)*†	1.3%(0.4%)	1.3%(1.0%)	1.1%(0.3%)
	Putamen	2.1%(3.2%)*†	1.1%(0.7%)	1.1%(0.7%)	1.0%(0.7%)	1.1%(0.7%)
[^18^F]Bavarostat (*n* = 14)	Caudate	2.0%(2.5%)*†	1.4%(1.1%)*†	1.5%(1.4%)*	1.3%(0.6%)*	1.1%(0.5%)
	Frontal	1.6%(1.7%)*†	0.5%(0.5%)*†	0.4%(0.6%)	0.3%(0.3%)	0.3%(0.2%)
[^18^F]FPEB (*n* = 9)	Caudate	6.5%(0.4%)*†	1.6%(1.6%)	1.5%(0.7%)	1.3%(0.9%)	1.3%(0.7%)
	Frontal	2.8%(1.2%)*†	1.4%(0.6%)*†	0.5%(0.5%)*	0.6%(0.3%)	0.4%(0.3%)
[^11^C]PBR28 (*n* = 3)	Insula	3.2%(0.7%)	1.9%(0.4%)	2.6%(0.3%)	1.8%(0.4%)	1.7%(0.5%)

Values are the median (interquartile range) of the %SD values around the fitted time-activity curves. * indicates statistically significant (*p* < 0.05) higher SD (i.e., poorer motion correction) than Vicra by single-tail Wilcoxon signed-rank test. † indicates significant (*p* < 0.05) higher SD than Optimized UMT by single-tail Wilcoxon signed-rank test. Statistical tests were not performed for [^11^C]PBR28 due to the small sample size. NMC: No Motion Correction. PRR: Post-reconstruction registration. SD: Standard deviation. UMT: United Imaging Healthcare Motion Tracking system

**Table 4:** Time-activity curve fitting residual SD for two NeuroEXPLORER [^18^F]FE-PE2I scans

Tracer	[^18^F]FE-PE2I (*n* = 2)			
Brain ROI	NMC	PRR	Baseline UMT	Optimized UMT
Caudate	3.0%, 3.7%	1.2%, 5.8%	1.1%, 1.2%	0.9%, 1.2%
Putamen	2.6%, 5.2%	1.3%, 2.5%	0.5%, 0.8%	0.4%, 0.7%
Ventral striatum	3.4%, 7.6%	1.7%, 4.5%	1.6%, 3.5%	2.1%, 3.2%
Striatum Average	2.7%, 5.0%	1.2%, 4.0%	0.8%, 1.5%	0.8%, 1.5%

Values are shown from two studies, one with moderate motion and one with large motion. The Striatum Average was computed by first averaging the SUV across the three striatal ROIs at each time point to form a TAC, after which the residual SD of the fitted TAC was calculated. NMC: No Motion Correction. PRR: Post-reconstruction registration. SD: Standard deviation. TAC: Time-Activity Curve. UMT: United Imaging Healthcare Motion Tracking system

## Supplementary Information

Below is the link to the electronic supplementary material.

Supplementary Material 1

## Data Availability

The data cannot be made publicly available upon publication because they contain sensitive personal information. The data that support the findings of this study are available upon reasonable request from the authors.

## References

[1] Li H, Badawi RD, Cherry SR, Fontaine K, He L, Henry S, et al. Performance characteristics of the NeuroEXPLORER, a next-generation human brain PET/CT imager. J Nucl Med. 2024;65:1320–6. 10.2967/jnumed.124.267767. 38871391 10.2967/jnumed.124.267767 PMC11294061

[2] Omidvari N, Shanina E, Leung EK, Sun X, Li Y, Mulnix T, et al. Quantitative accuracy assessment of the NeuroEXPLORER for diverse imaging applications: moving beyond standard evaluations. J Nucl Med. 2025;66:150–7. 10.2967/jnumed.124.268309. 39638433 10.2967/jnumed.124.268309 PMC11705792

[3] Lu Y, Naganawa M, Toyonaga T, Gallezot JD, Fontaine K, Ren S, et al. Data-driven motion detection and event-by-event correction for brain PET: comparison with Vicra. J Nucl Med. 2020;61:1397–403. 10.2967/jnumed.119.235515. 32005770 10.2967/jnumed.119.235515 PMC7456171

[4] Tellmann L, Fulton R, Pietrzyk U, Nickel I, Stangier I, Winz O, et al. Concepts of registration and correction of head motion in positron emission tomography. Z Med Phys. 2006;16:67–74. 10.1078/0939-3889-00293. 16696372 10.1078/0939-3889-00293

[5] Spangler-Bickell MG, Hurley SA, Deller TW, Jansen F, Bettinardi V, Carlson M, et al. Optimizing the frame duration for data-driven rigid motion estimation in brain PET imaging. Med Phys. 2021;48:3031–41. 10.1002/mp.14889. 33880778 10.1002/mp.14889 PMC9261293

[6] Revilla EM, Gallezot JD, Naganawa M, Toyonaga T, Fontaine K, Mulnix T, et al. Adaptive data-driven motion detection and optimized correction for brain PET. NeuroImage. 2022;252:119031. 10.1016/j.neuroimage.2022.119031. 35257856 10.1016/j.neuroimage.2022.119031 PMC9206767

[7] Lieffrig EV, Zeng T, Zhang J, Fontaine K, Fang X, Revilla E, et al. Multi-task deep learning and uncertainty estimation for PET head motion correction. In: IEEE 20th international symposium on biomedical imaging (ISBI); 2023. 10.1109/isbi53787.2023.10230791 10.1109/isbi53787.2023.10230791 PMC10725741 38111738

[8] Zeng T, Zhang J, Lieffrig EV, Cai Z, Chen F, You C, et al. Fast reconstruction for deep learning PET head motion correction. In: International conference on medical image computing and computer-assisted intervention, vol. 14229; 2023. pp. 710–719. 10.1007/978-3-031-43999-5_67. 10.1007/978-3-031-43999-5_67 PMC10758999 38174207

[9] Reimers E, Cheng JC, Sossi V. Deep-learning-aided intraframe motion correction for low-count dynamic brain PET. IEEE Trans Radiat Plasma Med Sci. 2024;8:53–63. 10.1109/Trpms.2023.3333202.

[10] Cai Z, Zeng T, Zhang J, Lieffrig ÉV, Fontaine K, You C, et al. PET head motion estimation using supervised deep learning with attention. IEEE Trans Med Imaging. 2026;45:1265-78. 10.1109/TMI.2025.3620714 10.1109/TMI.2025.3620714 PMC13097669 41082441

[11] Jin X, Mulnix T, Gallezot JD, Carson RE. Evaluation of motion correction methods in human brain PET imaging–a simulation study based on human motion data. Med Phys. 2013;40:102503. 10.1118/1.4819820. 24089924 10.1118/1.4819820 PMC3785538

[12] Bloomfield PM, Spinks TJ, Reed J, Schnorr L, Westrip AM, Livieratos L, et al. The design and implementation of a motion correction scheme for neurological PET. Phys Med Biol. 2003;48:959–78. 10.1088/0031-9155/48/8/301. 12741495 10.1088/0031-9155/48/8/301

[13] Chemli Y, Tetrault MA, Marin T, Normandin MD, Bloch I, El Fakhri G, et al. Super-resolution in brain positron emission tomography using a real-time motion capture system. NeuroImage. 2023;272:120056. 10.1016/j.neuroimage.2023.120056. 36977452 10.1016/j.neuroimage.2023.120056 PMC10122782

[14] Tan W, Wang Z, Zeng X, Boccia A, Wang X, Li Y, et al. High-resolution motion compensation for brain PET imaging using real-time electromagnetic motion tracking. Med Phys. 2025;52:201–18. 10.1002/mp.17437. 39422495 10.1002/mp.17437 PMC11716701

[15] Zeng T, Lu Y, Jiang W, Zheng J, Zhang J, Gravel P, et al. Markerless head motion tracking and event-by-event correction in brain PET. Phys Med Biol. 2023;68. 10.1088/1361-6560/ad0e37 10.1088/1361-6560/ad0e37 PMC10713921 37983915

[16] Olesen OV, Sullivan JM, Mulnix T, Paulsen RR, Hojgaard L, Roed B, et al. List-mode PET motion correction using markerless head tracking: proof-of-concept with scans of human subject. IEEE Trans Med Imaging. 2013;32:200–9. 10.1109/TMI.2012.2219693. 23008249 10.1109/TMI.2012.2219693

[17] Iwao Y, Akamatsu G, Tashima H, Takahashi M, Yamaya T. Marker-less and calibration-less motion correction method for brain PET. Radiol Phys Technol. 2022;15:125–34. 10.1007/s12194-022-00654-6. 35239130 10.1007/s12194-022-00654-6

[18] Kyme AZ, Se S, Meikle SR, Fulton RR. Markerless motion estimation for motion-compensated clinical brain imaging. Phys Med Biol. 2018;63:105018. 10.1088/1361-6560/aabd48. 29637899 10.1088/1361-6560/aabd48

[19] Jin X, Chan C, Mulnix T, Panin V, Casey ME, Liu C, et al. List-mode reconstruction for the Biograph mCT with physics modeling and event-by-event motion correction. Phys Med Biol. 2013;58:5567–91. 10.1088/0031-9155/58/16/5567 23892635 10.1088/0031-9155/58/16/5567 PMC3835774

[20] Montgomery AJ, Thielemans K, Mehta MA, Turkheimer F, Mustafovic S, Grasby PM. Correction of head movement on PET studies: comparison of methods. J Nucl Med. 2006;47:1936–44. 17138736

[21] Serafin J, Grisetti G. Using extended measurements and scene merging for efficient and robust point cloud registration. Robot Auton Syst. 2017;92:91–106. 10.1016/j.robot.2017.03.008.

[22] Steinbrücker F, Sturm J, Cremers D. Volumetric 3D Mapping in real-time on a CPU. In: IEEE international conference on robotics and automation (ICRA); 2014. pp. 2021–2028.

[23] Scarpazza DP, Villa O, Petrini F. Efficient breadth-first search on the Cell/BE processor. IEEE Trans Parallel Distrib syst. 2008;19:1381–95. 10.1109/Tpds.2007.70811.

[24] Jakoby BW, Bercier Y, Conti M, Casey ME, Bendriem B, Townsend DW. Physical and clinical performance of the mCT time-of-flight PET/CT scanner. Phys Med Biol. 2011;56:2375–89. 10.1088/0031-9155/56/8/004. 21427485 10.1088/0031-9155/56/8/004

[25] Sun C, Revilla EM, Zhang J, Fontaine K, Toyonaga T, Gallezot JD, et al. An objective evaluation method for head motion estimation in PET-motion corrected centroid-of-distribution. NeuroImage. 2022;264:119678. 10.1016/j.neuroimage.2022.119678. 36261057 10.1016/j.neuroimage.2022.119678

[26] Naganawa M, Nabulsi N, Henry S, Matuskey D, Lin SF, Slieker L, et al. First-in-human assessment of ^11^C-LSN3172176, an M1 muscarinic acetylcholine receptor PET radiotracer. J Nucl Med. 2021;62:553–60. 10.2967/jnumed.120.246967. 32859711 10.2967/jnumed.120.246967 PMC8049371

[27] Koole M, Van Weehaeghe D, Serdons K, Herbots M, Cawthorne C, Celen S, et al. Clinical validation of the novel HDAC6 radiotracer [^18^F]EKZ-001 in the human brain. Eur J Nucl Med Mol Imaging. 2021;48:596–611. 10.1007/s00259-020-04891-y. 32638097 10.1007/s00259-020-04891-y PMC7835181

[28] Wong DF, Waterhouse R, Kuwabara H, Kim J, Brasic JR, Chamroonrat W, et al. 18F-FPEB, a PET radiopharmaceutical for quantifying metabotropic glutamate 5 receptors: a first-in-human study of radiochemical safety, biokinetics, and radiation dosimetry. J Nucl Med. 2013;54:388–96. 10.2967/jnumed.112.107995. 23404089 10.2967/jnumed.112.107995 PMC9911749

[29] Sabri O, Becker GA, Meyer PM, Hesse S, Wilke S, Graef S, et al. First-in-human PET quantification study of cerebral alpha4beta2* nicotinic acetylcholine receptors using the novel specific radioligand (-)-[(18)F]Flubatine. NeuroImage. 2015;118:199–208. 10.1016/j.neuroimage.2015.05.065. 26037057 10.1016/j.neuroimage.2015.05.065

[30] Fujita M, Imaizumi M, Zoghbi SS, Fujimura Y, Farris AG, Suhara T, et al. Kinetic analysis in healthy humans of a novel positron emission tomography radioligand to image the peripheral benzodiazepine receptor, a potential biomarker for inflammation. NeuroImage. 2008;40:43–52. 10.1016/j.neuroimage.2007.11.011. 18093844 10.1016/j.neuroimage.2007.11.011 PMC2265774

[31] Jenkinson M, Bannister P, Brady M, Smith S. Improved optimization for the robust and accurate linear registration and motion correction of brain images. NeuroImage. 2002;17:825–41. 10.1016/s1053-8119(02)91132-8. 12377157 10.1016/s1053-8119(02)91132-8

[32] Tzourio-Mazoyer N, Landeau B, Papathanassiou D, Crivello F, Etard O, Delcroix N, et al. Automated anatomical labeling of activations in SPM using a macroscopic anatomical parcellation of the MNI MRI single-subject brain. NeuroImage. 2002;15:273–89. 10.1006/nimg.2001.0978. 11771995 10.1006/nimg.2001.0978

[33] Dahnke R, Yotter RA, Gaser C. Cortical thickness and central surface estimation. NeuroImage. 2013;65:336–48. 10.1016/j.neuroimage.2012.09.050. 23041529 10.1016/j.neuroimage.2012.09.050

[34] Munk OL, Rodell AB, Danielsen PB, Madsen JR, Sorensen MT, Okkels N, et al. Validation of a data-driven motion-compensated PET brain image reconstruction algorithm in clinical patients using four radiotracers. EJNMMI Phys. 2025;12:11. 10.1186/s40658-025-00723-w. 39899171 10.1186/s40658-025-00723-w PMC11790531

[35] Neri I, Spangler-Bickell M, Fallanca F, Gattuso G, Barbera M, Ghezzo S, et al. Data-driven motion correction of ^11^C-methionine PET images on a cohort of pediatric patients with brain tumor in a PET/MRI study. IEEE Trans Radiat Plasma Med Sci. 2025;10:240–48. 10.1109/TRPMS.2025.3568136

[36] Aziz R, Muller FM, Withofs N, Maebe J, Dadgar M, Vervenne B, et al. Motion study on upright patient positioning in walk-through PET: evaluation in a clinical setting. EJNMMI Phys. 2025;12:98. 10.1186/s40658-025-00815-7. 41307633 10.1186/s40658-025-00815-7 PMC12660553

[37] Sasaki T, Ito H, Kimura Y, Arakawa R, Takano H, Seki C, et al. Quantification of dopamine transporter in human brain using PET with 18F-FE-PE2I. J Nucl Med. 2012;53:1065–73. 10.2967/jnumed.111.101626. 22689927 10.2967/jnumed.111.101626

