# Supplementary material for: Optimization of brain PET markerless head motion correction in a large human cohort
Source: EJNMMI Phys. 2026 May 29;13:76. doi: 10.1186/s40658-026-00885-1 (PMC13433684; doi:10.1186/s40658-026-00885-1)
Supplement: Supplementary file 1 [file 40658_2026_885_MOESM1_ESM.docx]

**Supplemental material**

## Morphological closing operation:

To fill small holes in the UMT depth image, we utilized the morphological closing operation through OpenCV. This operation involved two steps of dilations followed by erosions, using a 10 by 10 kernel with 3-mm voxel size. The outcome of this operation is depicted in Supplemental Fig. 1. In Supplemental Fig. 1a, the original depth image exhibited fragmentation due to the UMT angle and eye socket effects, resulting in data gaps (denoted in the blue frame) dividing the face area into multiple regions. After application of the closing operator (Supplemental Fig. 1b), a contiguous face area was delineated.

## Vicra motion data pre-assessment:

## To avoid potential biases resulting from incorrect Vicra data (e.g., due to slippage or wobbling of the cap holding the Vicra tool), we utilized an objective evaluation method for motion correction called Motion Corrected Centroid-of-Distribution (MCCOD) [1] to assess the Vicra motion data and remove questionable data. If motion data are accurate, the MCCOD traces are expected to be smoothly varying, where any change is due to tracer redistribution. The Vicra MCCOD curves were evaluated by two observers, and eight studies with data that were considered problematic by both experts were excluded. Supplemental Fig. 2 shows one example of a high quality Vicra MCCOD curve ([^18^F]Bavarostat) and two examples of inaccurate Vicra motion correction (one [^18^F]FPEB and one [^18^F]Bavarostat). In Supplemental Fig. 2b, the magnitude of step motions in the uncorrected COD-x trace was reduced in the MCCOD-x tracers, but residual displacements were observed. In Supplemental Fig. 2c, the trend in the COD-y curve starting at 60 min was altered but not removed in the MCCOD-y curve. Consequently, these data were not considered to be appropriate to use as a gold standard and were omitted from the image analysis and quantitative comparison.

## Motion data selection in simulation study:

To investigate the effect of uncorrected or inaccurate motion in a simulation study (Simulation 1: Large rigid motion), we identified 4 distinct periods of substantial motion from Vicra human data. The magnitude of intraframe motion for each frame was calculated as twice the standard deviation of motion for any point within the field of view (FOV), following the methodology in [2]. Specifically, eight points were selected as the vertices of a 10-cm cube centered in the scanner FOV, and the average motion across these points was considered as the intraframe motion. The scan-level motion magnitude was then computed as the average of intraframe motions across all frames, weighted by the number of events in each frame, ensuring that frames with higher counts contribute proportionally more to the overall metric. According to findings in [2], motion magnitudes exceeding 2.8 mm represented the most extreme 15% of motion in over 500 PET studies. Consequently, we chose four 5-min frames from four different subjects with an average intraframe motion magnitude of 3.5±0.4 mm.

## In the second simulation study designed to investigate the effects of facial expressions, the experimental protocol for acquiring motion data was identical for both volunteers, with the same planned sequence of facial expressions and rigid motion. While the actual motion naturally varied between volunteers, the protocol ensured comparable conditions for evaluation. Using the same procedure for calculating motion magnitude, the average intraframe motion magnitude across the four acquisitions was 17.7±2.7 mm.

## UMT video:

## To visualize UMT data and gather information on facial motion and head movements retrospectively, a video technology was developed. During the UMT data acquisition process, the stereovision camera captured depth images through structured light. These depth images enabled the calculation of the spatial point clouds [3]. In addition, using the OpenCV library [4], a UMT video was generated by combining a sequence of depth images, acquired at 10 frames per second, and including embed time of day and frame index information. Supplemental Fig. 4 illustrates 3 different frames extracted from a UMT video. The volunteer provided consent for the use of the video frames.

## Simulation methodology:

At each millisecond interval timestamp, a random selection of lines of response (LORs) was chosen. The end-points of each LOR were moved using the transformation matrix generated by the Vicra at that time [2]. These transformed LORs were forward projected through the ground truth activity image, following the methodology in [5]. Random and scattered events were not included in the simulation process. Ultimately, the simulated listmode files contained 200 million true events.

# Reference:

1.	Sun C, Revilla EM, Zhang J, Fontaine K, Toyonaga T, Gallezot JD, et al. An objective evaluation method for head motion estimation in PET-Motion corrected centroid-of-distribution. Neuroimage. 2022;264:119678. doi:10.1016/j.neuroimage.2022.119678.

2.	Jin X, Mulnix T, Gallezot JD, Carson RE. Evaluation of motion correction methods in human brain PET imaging--a simulation study based on human motion data. Med Phys. 2013;40:102503. doi:10.1118/1.4819820.

3.	Poranne R, Tarini M, Huber S, Panozzo D, Sorkine-Hornung O. Autocuts: Simultaneous Distortion and Cut Optimization for UV Mapping. Acm T Graphic. 2017;36. doi:Artn 21510.1145/3130800.3130845.

4.	Bradski G. The OpenCV library. Dr Dobbs J. 2000;25:120-+.

5.	Germino M, Carson RE. Cardiac-gated parametric images from (82) Rb PET from dynamic frames and direct 4D reconstruction. Med Phys. 2018;45:639-54. doi:10.1002/mp.12710.

**Supplemental Table 1a** Bias in static brain ROI analysis of [^18^F]Bavarostat studies (n=14)

| Tracer | [^18^F]Bavarostat | | | |
| --- | --- | --- | --- | --- |
| Brain ROI | NMC | PRR | Baseline UMT | Optimized UMT |
| Amygdala | -5.3%±7.7% | -1.5%±1.4% | 0.2%±1.9% | -0.3%±1.3% |
| Caudate | -10.9%±8.7% | -2.7%±1.6% | -0.6%±3.2% | -0.6%±1.5% |
| Cerebellum Cortex | -4.8%±5.4% | -1.5%±2.9% | 1.6%±3.4% | 0.9%±4.0% |
| Frontal | -12.1%±6.7% | -2.3%±2.2% | -0.1%±2.5% | -0.2%±1.5% |
| Hippocampus | -2.9%±4.6% | -1.6%±1.3% | -0.5%±2.8% | -0.2%±1.7% |
| Insula | -5.9%±3.6% | -1.7%±1.7% | 0.3%±0.9% | 0.3%±0.9% |
| Occipital | -3.2%±3.7% | -1.7%±3.1% | 0.9%±2.5% | 0.7%±4.0% |
| Parietal | -5.5%±4.7% | -1.9%±2.6% | -0.9%±3.7% | -0.3%±2.8% |
| Putamen | -7.0%±4.1% | -2.2%±1.6% | -0.2%±0.9% | -0.3%±1.1% |
| Temporal | -8.2%±5.7% | -1.6%±1.5% | 0.8%±1.3% | 0.7%±1.7% |
| Thalamus | -5.1%±4.2% | -2.3%±2.4% | -0.6%±1.7% | -0.3%±1.9% |
| GM Average±SD | -6.5%±4.1% | -1.9%±1.5% | 0.1%±1.3% | 0.0%±1.2% |

## Supplemental Table 1b Bias in static brain ROI analysis of [^18^F]FPEB studies (n=9)

| Tracer | [^18^F]FPEB | | | |
| --- | --- | --- | --- | --- |
| Brain ROI | NMC | PRR | Baseline UMT | Optimized UMT |
| Amygdala | -9.7%±9.1% | -3.5%±4.6% | -1.3%±3.8% | -0.6%±2.2% |
| Caudate | -19.6%±11.3% | -4.4%±3.7% | -0.7%±3.1% | -1.8%±4.1% |
| Cerebellum Cortex | -3.7%±4.8% | -3.6%±3.1% | 0.5%±2.8% | -1.3%±4.7% |
| Frontal | -13.1%±6.8% | -3.1%±2.5% | -1.4%±3.3% | -1.4%±3.0% |
| Hippocampus | -7.4%±5.4% | -2.5%±3.9% | -2.3%±4.7% | -1.5%±4.3% |
| Insula | -10.4%±5.5% | -2.6%±1.8% | 0.1%±1.2% | 0.3%±1.9% |
| Occipital | -4.2%±7.6% | -1.5%±2.0% | -0.9%±2.7% | -0.5%±3.0% |
| Parietal | -6.6%±6.4% | -1.0%±1.1% | -1.5%±3.0% | -1.1%±3.2% |
| Putamen | -7.0%±5.0% | -2.5%±1.3% | 0.0%±1.4% | -0.7%±2.6% |
| Temporal | -7.1%±5.2% | -1.4%±1.5% | -1.0%±3.8% | -0.6%±3.2% |
| Thalamus | -8.0%±5.1% | -3.0%±2.3% | -0.9%±1.9% | -1.1%±2.2% |
| GM Average±SD | -9.2%±6.0% | -2.6%±2.0% | -0.9%±2.6% | -0.9%±2.5% |

**Supplemental Table 1c** Bias in static brain ROI analysis of [^18^F]NCFHEB studies (n=7)

| Tracer | [^18^F]NCFHEB | | | |
| --- | --- | --- | --- | --- |
| Brain ROI | NMC | PRR | Baseline UMT | Optimized UMT |
| Amygdala | -1.3%±1.8% | -0.7%±1.1% | 0.3%±0.7% | 0.2%±0.7% |
| Caudate | -5.0%±4.9% | -1.2%±2.5% | -0.3%±1.5% | 0.4%±1.3% |
| Cerebellum Cortex | -2.8%±3.4% | -0.6%±2.6% | 0.5%±1.7% | 0.1%±1.7% |
| Frontal | -7.5%±3.8% | -1.0%±1.8% | -0.1%±0.4% | 0.1%±0.5% |
| Hippocampus | 1.6%±4.1% | 0.2%±1.3% | 0.3%±1.3% | -0.4%±0.8% |
| Insula | -0.5%±1.1% | -0.5%±0.7% | 0.0%±0.4% | 0.0%±0.2% |
| Occipital | -0.4%±2.3% | 2.0%±1.6% | 0.8%±1.4% | 0.2%±1.8% |
| Parietal | -1.9%±4.7% | 1.1%±2.3% | 0.1%±1.0% | 0.4%±1.2% |
| Putamen | -0.8%±1.5% | -0.6%±0.8% | 0.1%±0.4% | 0.0%±0.2% |
| Temporal | -4.1%±2.7% | -0.3%±1.0% | 0.1%±0.6% | -0.1%±0.4% |
| Thalamus | -4.4%±3.6% | 0.0%±0.6% | 0.5%±0.6% | 0.2%±0.5% |
| GM Average±SD | -2.7%±1.9% | -0.2%±0.7% | 0.2%±0.4% | 0.1%±0.2% |

**Supplemental Table 1d** Bias in static brain ROI analysis of [^11^C]PBR28 studies (n=3)

| Tracer | [^11^C]PBR28 | | | |
| --- | --- | --- | --- | --- |
| Brain ROI | NMC | PRR | Baseline UMT | Optimized UMT |
| Amygdala | -0.0%±1.1% | -0.1%±0.8% | 0.4%±0.9% | 0.4%±1.1% |
| Caudate | -12.3%±8.0% | -1.9%±2.3% | 0.3%±0.9% | 0.8%±1.5% |
| Cerebellum Cortex | -3.2%±1.8% | -2.1%±1.2% | 0.2%±1.5% | 0.0%±1.2% |
| Frontal | -7.5%±4.7% | -0.1%±1.2% | 0.6%±1.7% | 0.9%±1.8% |
| Hippocampus | 0.4%±0.4% | 1.0%±0.5% | 0.2%±1.0% | 0.0%±0. 8% |
| Insula | -4.8%±3.2% | -0.2%±0.5% | 0.2%±0.2% | 0.3%±0.2% |
| Occipital | 1.2%±0.7% | 2.5%±2.2% | 0.4%±0.9% | -0.1%±0.8% |
| Parietal | 1.7%±2.1% | 0.7%±1.2% | 0.7%±0.4% | 0.4%±0.8% |
| Putamen | -3.8%±4.1% | -0.3%±0.5% | -0.3%±0.3% | 0.0%±0.7% |
| Temporal | -1.9%±2.0% | 0.3%±0.3% | 0.5%±0.7% | 0.6%±0.5% |
| Thalamus | -2.3%±2.0% | -0.8%±1.0% | -0.4%±0.4% | -0.7%±0.4% |
| GM Average±SD | -2.9%±1.9% | -0.1%±0.7% | 0.2%±0.7% | 0.2%±0.8% |

ROI bias values shown as average ± standard deviation of the % difference with respect to Vicra values across all subjects. To calculate GM Average and GM SD, SUVs were first averaged across all GM ROIs for each subject to obtain a subject-level GM SUV. Percent differences relative to Vicra were then calculated at the subject level and summarized across subjects. NMC: No Motion Correction. PRR: Post-reconstruction registration. ROI: region-of-interest. SD: standard deviation. SUV: Standardized Uptake Values. UMT: United Imaging Healthcare Motion Tracking system

**Supplemental Fig. 1** Original depth image (a) and outcome after the morphological closing operation (b). The color indicates varying distances from the camera. The black area represents invalid or undefined depth data

**Supplemental Fig. 2** Blue curves show the uncorrected centroid of distribution (COD) traces. Orange curves show the motion-corrected COD (MCCOD) tracers. (a) One typical high-quality Vicra MCCOD curve. (b,c) two problematic Vicra MCCOD curves which showed uncorrected motion. These datasets were excluded from the comparison to the UMT camera data

**Supplemental Fig. 3** Ideal activity images derived from a human MR image utilized in the simulation study. The activity levels are set as follows: grey matter: 4, white matter:1, and cerebrospinal fluid:0

**Supplemental Fig. 4** Different frames from the UMT video. No facial expression (a), eye rubbing (b), and eyebrow movement (c) are shown

**Supplemental Fig. 5** Mean (top) and SD (bottom) across small ROIs of the percent difference of NMC and 3 MC methods versus Vicra over individual scan frames for the (a) [^18^F]Bavarostat study (n=14), (b) [^18^F]FPEB study (n=9), (c) [^18^F]NCFHEB study (n=7) and (d) [^11^C]PBR28 study (n=3). NMC: No Motion Correction. PRR: Post-reconstruction registration. SD: Standard deviation. UMT: United Imaging Healthcare Motion Tracking system

## Supplemental Fig. 6 Examples of caudate (volume 7.4 mL) TAC and fitting of data with different motion corrections from two [^11^C]LSN3172176 scans with typical motion (a) and extreme motion (b). For the extreme motion case, the percent SD of the residuals around the fitting curve indicates higher variability in the results of NMC, PRR and Baseline UMT compared to Optimized UMT and Vicra. NMC: No Motion Correction. PRR: Post-reconstruction registration. SD: standard deviation. TAC: time activity curve. UMT: United Imaging Healthcare Motion Tracking system

## Supplemental Fig. 7 UMT motion data from 30 to 90 minutes postinjection for subjects with moderate motion (a) and large motion (b). UMT: United Imaging Healthcare Motion Tracking system
